# ASPM Induces Radiotherapy Resistance by Disrupting Microtubule Stability Leading to Chromosome Malsegregation in Non‐Small Cell Lung Cancer

**DOI:** 10.1002/EXP.20230024

**Published:** 2025-05-07

**Authors:** Tao Zhong, Ning Liu, Juan Wang, Songbo Xie, Lisheng Liu, Minglei Wang, Fei Wu, Xiaozheng Chen, Changyan Xiao, Xiaoxiao Gongye, Meng Wu, Liewei Wen, Jinming Yu, Dawei Chen

**Affiliations:** ^1^ Institute of Immunology and Molecular Medicine Shandong Provincial Key Laboratory of Cell Biomedical Technology College of Basic Medicine Jining Medical University Jining Shandong China; ^2^ Research Unit of Radiation Oncology Chinese Academy of Medical Sciences Jinan Shandong China; ^3^ Department of Radiation Oncology and Shandong Provincial Key Laboratory of Radiation Oncology Shandong Cancer Hospital and Institute Shandong First Medical University and Shandong Academy of Medical Sciences Jinan Shandong China; ^4^ School of Life Sciences and Medicine Shandong University of Technology Zibo Shandong China; ^5^ Guangdong Provincial Key Laboratory of Tumor Interventional Diagnosis and Treatment Zhuhai People's Hospital (The Affiliated Hospital of Beijing Institute of Technology) Beijing Institute of Technology Zhuhai China

**Keywords:** ASPM, chromosomal malsegregation, radiotherapy, radiotherapy resistance

## Abstract

Radiotherapy (RT) resistance remains a substantial challenge in cancer therapy. Although physical factors are optimizing, the biological mechanisms for RT resistance are still elusive. Herein, we explored potential reasons for this difficult problem by generating RT‐resistant models for in vitro and in vivo experiments. We found that abnormal spindle‐like microcephaly‐associated protein (ASPM) was highly expressed in RT‐resistant samples and significantly correlated with disease advance in lung adenocarcinoma. Mechanistically, ASPM helps RT‐resistant cells to evade spindle checkpoint surveillance and complete cell division after irradiation through destruction of microtubule stability, with subsequent increases in chromosome mis‐segregation and deteriorating chromosomal stability during mitosis. Depletion of ASPM stabilized microtubules and significantly decreased chromosome mis‐segregation, restoring the sensitivity of RT‐resistant cells to radiation. We further found, with bioinformatics analysis, amino acid sequence 963–1263 of ASPM as a potential new drug target for overcoming RT resistance and identified 9 drug pockets within this domain for clinical translation. Our findings suggest that ASPM is a key regulator with an important role in promoting RT resistance in non‐small cell lung cancer, and that suppressing or blocking its expression could be worth exploring as therapy for a variety of RT‐resistant cancers.

## Graphical abstract

Abnormal spindle‐like microcephaly‐associated protein helps radiotherapy resistant cells to evade spindle checkpoint surveillance and complete cell division after irradiation through destruction of microtubule stability, with subsequent increases in chromosome mis‐segregation and deteriorating chromosomal stability during mitosis to promote radiotherapy resistant in non‐small cell lung cancer.

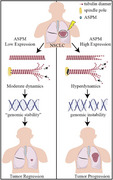

## 1 Introduction

Radiotherapy (RT) is one of the main modes of treatment for non‐small cell lung cancer (NSCLC) [1], with about 60% of all newly diagnosed cases requiring RT as first‐line treatment [2]. Nevertheless, NSCLC in some patients is resistant to RT and is unlikely to respond to this treatment. Previous studies of RT resistance primarily focused on tumor‐cell killing mediated by immune cells [3], but changes in the tumor cells themselves that render resistance to RT have not been fully revealed. Moreover, validated biomarkers for response to RT in NSCLC are lacking [4], and as such overtreatment with RT in RT‐resistant disease contributes an unnecessary burden and contributes to treatment‐induced morbidity [5]. Thus, additional information is needed on the molecular regulators of sensitivity to radiation, with detailed mechanistic studies to dissect the complexities of these interactions.

Many characteristics of cancer cells are lost or perturbed in RT‐resistant cancer cells [6], including tumor‐cell survival after RT that may be related to improper segregation of chromosomes [7]. Defects in chromosome segregation leads to aneuploidy, which in turn leads to genomic instability via defects in mitosis, which is associated with disease progression [8]. Genomic instability is thought to increase the likelihood of generating combinations of chromosomes that confer a selective growth advantage [9]. Although mitotic cells have ways of ensuring correct segregation of chromosomes, those pathways may not be fully operational in RT‐resistant tumor cells [10]. Intrigued by this, we wanted to know whether and how these RT‐resistant cancer cells achieve incorrect chromosome segregation.

Although a growing body of evidence underscores the importance of cytokinesis in chromosome segregation [11], whether cytokinesis has a significant role in the biology of RT resistance is still an open question. Cytokinesis causes the physical separation of the cytoplasm of a mother cell into two daughter cells, with each having the correct complement of chromosomes. This process depends on having a stable microtubule cytoskeleton and correspondingly on the integrity and function of the spindle complex [12]. Stable microtubules form a structurally symmetric spindle about metaphase‐plate‐positioned chromosomes during mitosis [13]. Essential to the chromosomal segregation process is that all chromosomes bi‐orient on the mitotic spindle so that the two daughter cells receive equal sets of chromosomes as they divide. A cell model that can recapitulate RT resistance would be quite useful for functional investigations of cytokinesis in RT and resistance to clarify the contribution of RT resistance to disease progression.

In the present study, we report an RT‐resistant NSCLC model with significant chromosomal segregation defects consistent with the results we found in human RT‐resistant NSCLC. Specifically, we found that disordered levels of abnormal spindle‐like microcephaly‐associated protein (ASPM) can enhance RT resistance by affecting microtubule stability and increasing chromosome mis‐segregation. Under physiological conditions, these RT‐resistant cells can evade spindle checkpoint surveillance and complete cell division after irradiation. Mechanistically, the dynamics of intracellular microtubule cytoskeleton formation and stability were disrupted, leading to spindle misorientation in mitotic cells that promoted cell survival by increasing chromosomal mis‐segregation. We further identified, using bioinformatics analysis, that the amino acid sequence 963–1263 of ASPM could be an effective drug target to overcome RT resistance, and we found 9 drug pockets within this domain. Our findings revealed that chromosome mis‐segregation can directly regulate the viability of RT‐resistant cancer cells during RT treatment, which could have substantial implications for future treatments involving radiation.

## 2 Results

### 2.1 ASPM Expression in RT‐Resistant Cells Correlates With Disease Progression in Lung Adenocarcinoma

**FIGURE 1:**
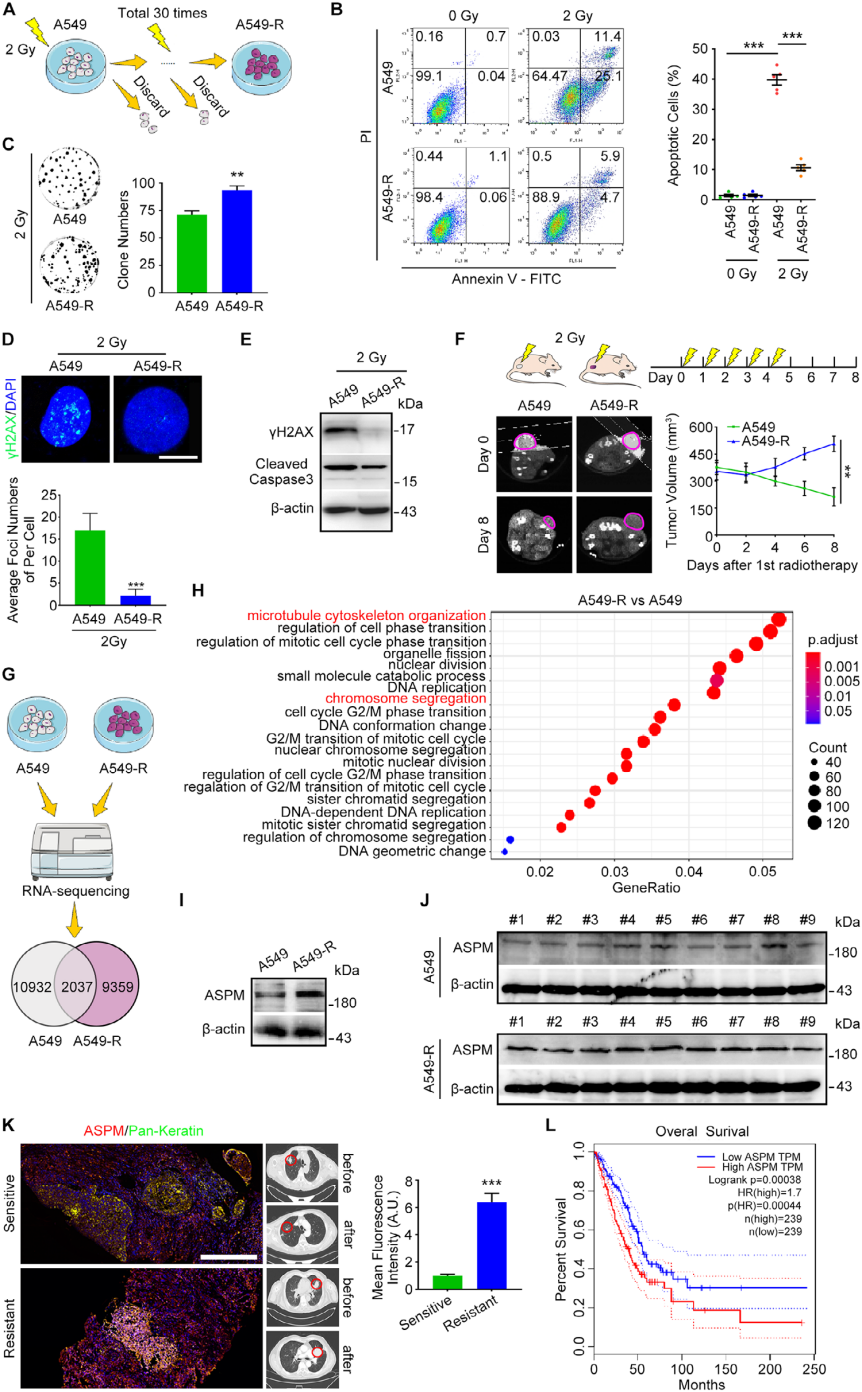
ASPM is highly expressed in RT‐resistant cells and tissues and is associated with poor survival in lung adenocarcinoma. (A) Schematic diagram showing the workflow of the creation of A549‐R cells in vitro. (B) Flow cytometry analysis of apoptotic cell percentages in A549 cells and A549‐R cells after irradiation to 2 Gy. The experiment was repeated five times in each group, *n* = 5. (C) Representative pictures showing analyses of numbers of A549 and A549‐R clones after 2 Gy irradiation. (D) Quantification of the generated average γH2AX foci numbers in A549 cells and A549‐R cells after 2 Gy treatment. Scale bar: 6 µm. *n* = 12 in each group. (E) Western blot showing the levels of γH2AX and cleaved caspase‐3 after 2 Gy irradiation. (F) Subcutaneous tumor volume formed by A549‐R cells in nude mice did not decrease after 2 Gy radiotherapy. There are 6 mice in each group, *n* = 6. (G) Schematic diagram showing the workflow of RNA sequencing and contrast. (H) Enriched pathways of differently expressed genes for A549‐R cells contrasted with A549 cells. (I,J) Western blot showing the levels of ASPM in A549 cells, A549‐R cells, and tumors formed by these cells after implantation in nude mice. The experiment was repeated three times. (K) Co‐staining of pan‐CK (green) + ASPM (red) shows high ASPM expression in biopsy samples from patients with RT‐resistant non‐small cell lung cancer (with resistance defined by the CT imaging results). Red circles represent tumor volume changes before and after RT. Samples from 2 patients in each group. Scale bar: 300 µm. Blue, DAPI. *n* = 3. (L) High expression of ASPM was associated with poorer survival in patients with lung adenocarcinoma in the Gene Expression Profiling Interactive Analysis database. NS, not significant; TPM, transcripts per million. **p < 0.01. ***p < 0.001.

To investigate the clinical phenomenon of radiation resistance in NSCLC, we first sought to create a system suitable for interrogation of contributors to RT resistance. Based on the guidelines for diagnosis and treatment of NSCLC of Chinese Society of Clinical Oncology (CSCO), patients with unresectable NSCLC, PS = 2, is recommended conventional radiotherapy (target dose [60–66] Gy/[30–33] frequency/[6–7] weeks) as the primary therapeutic regimen. The RTOG 0617 study showed that further increasing the total radiotherapy dose to 74 Gy did not improve efficacy [14].We exposed A549 cells to 30 rounds of 2 Gy, with stepwise selection of resistant cells (A549‐R) (Figure 1A). We then exposed the resistant cells to another 2 Gy and measured the ratio of apoptotic cells. As expected, the percentage of apoptotic cells decreased sharply in the A549‐R cells but significantly increased in A549 cells (Figure 1B). Similar results were presented in PC9 cells (Figure S1A). These results were confirmed by counting the number of A459 and A549‐R clones after the irradiation (Figure 1C). To extend our characterization of characteristics possibly related to RT resistance in A549‐R cells, we measured the expression of γH2AX and cleaved caspase‐3 in A549 and A549‐R cells after a 2‐Gy irradiation and found declines in both in the A549‐R cells (Figure 1D,E). We next injected A549 and A549‐R cells subcutaneously into the flanks of nude mice and tested the RT resistance of the resulting tumors in vivo. Irradiation of the implanted tumors to 2 Gy led to significant reduction in A549 tumor volumes but not the A549‐R tumors (Figure 1F). These in vivo results are consistent with our in vitro results and confirm the RT resistance in our A549‐R cell model.

As a next step in examining the molecular contributors to RT resistance in NSCLC, we compared mRNA expression in A549 cells and A549‐R cells (Figure 1G) and identified differences in genes associated with microtubule cytoskeleton organization and chromosome segregation (Figure 1H). These findings led us to consider ASPM for further study. ASPM expression levels in A549‐R cells in culture and in subcutaneous tumors in mice were higher than in A549 cells (Figure 1I,J). This result was verified with core biopsy samples of lung tissue from patients with known RT‐resistant NSCLC (with sensitivity vs. resistance defined by CT imaging findings) (Figure 1K). Next, we analyzed the Gene Expression Profiling Interactive Analysis (GEPIA) database for ASPM expression in relation to survival and found that patients with lung cancer and high ASPM expression had higher mortality rates than patients with low ASPM expression (Figure 1L). These findings prompted us to investigate APSM further for its possible involvement in regulating RT resistance in NSCLC.

### 2.2 ASPM Regulates Microtubule Stability in RT‐Resistant Cells

**FIGURE 2:**
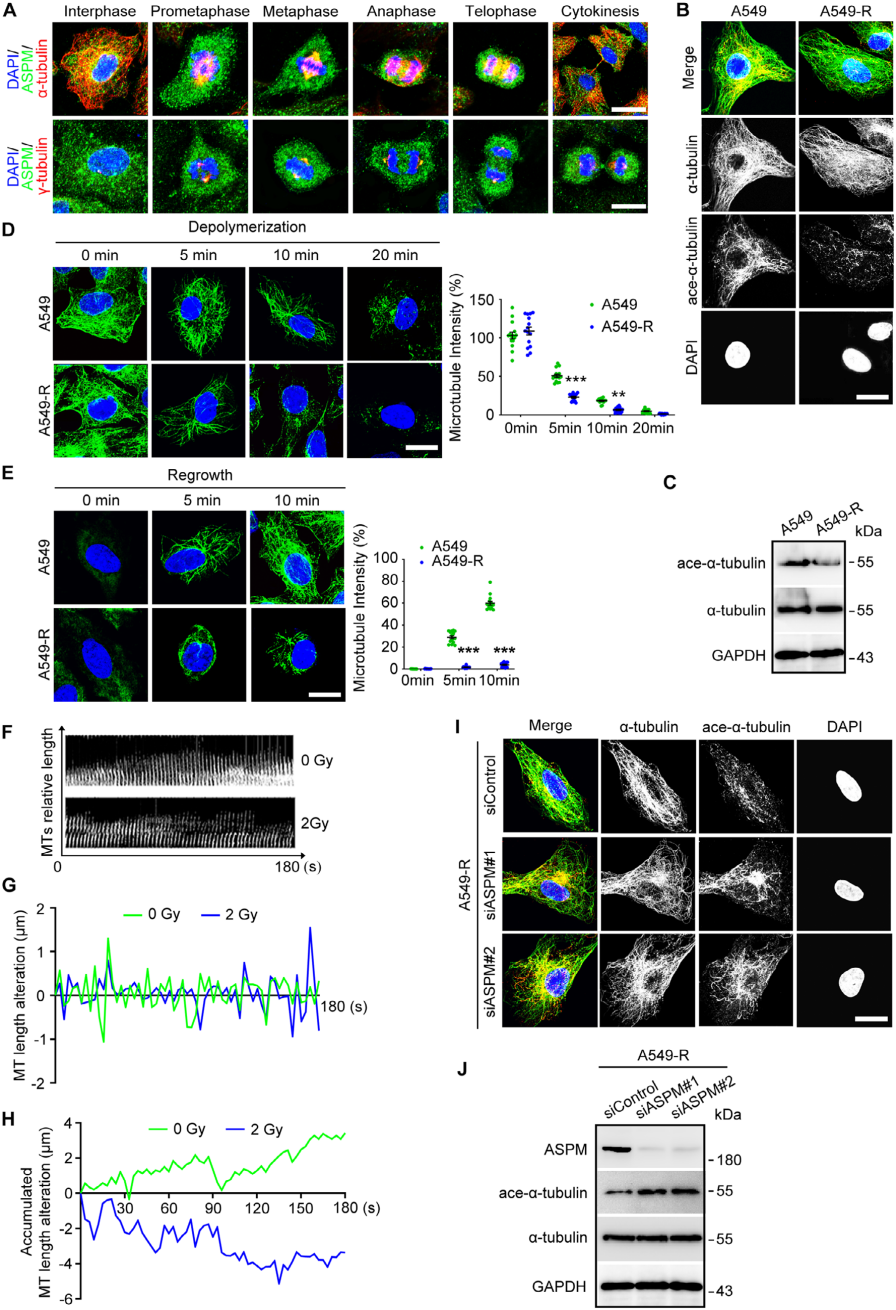
High levels of ASPM perturb the stability of microtubules in A549‐R cells. (A) Immunofluorescence staining using anti‐ASPM, anti‐α‐tubulin, anti‐γ‐tubulin, and DAPI in A549‐R cells to detect ASPM co‐localization with microtubules during mitosis. Scale bar: 12 µm. (B) Immunofluorescence images of A549 cells and A549‐R cells with anti‐α‐tubulin (green) and anti‐acetylated α‐tubulin (red) antibodies and DAPI (blue). Scale bar: 10 µm. (C) Immunoblots showing levels of acetylated α‐tubulin in A549 cells and A549‐R cells. (D) Immunofluorescence images of A549 cells and A549‐R cells incubated on ice for the indicated time, followed by staining with anti‐α‐tubulin antibodies (green) and DAPI (blue). Scale bar: 10 µm, with quantification of microtubule intensity in cells treated as described above at various times afterward. *n* ≥ 12 in each group. (E) Immunostaining with anti‐α‐tubulin antibodies (green) and DAPI (blue) in A549 cells and A549‐R cells incubated on ice for 45 min to induce complete microtubule disassembly and incubating at 37°C for the indicated times. Scale bar: 10 µm. The panel below shows quantification of microtubule intensity in cells treated as described above at various times afterward. *n* ≥ 6 in each group. (F) Time‐lapse images showing variations in microtubule length in HeLa cells stably expressing GFP‐tagged α‐tubulin over time after irradiation with 2 Gy. Quantification of changes in microtubule length (G) and accumulated changes in microtubule length (H) from panel F. (I) Immunostaining with anti‐acetylated α‐tubulin antibodies and DAPI in A549‐R cells transfected with control or *ASPM* siRNAs. Scale bar: 10 µm. (J) Immunoblots showing levels of ASPM, acetylated α‐tubulin, α‐tubulin, in A549‐R cells transfected with control or *ASPM* siRNAs. **p < 0.01. ***p < 0.001.

Previous studies have shown that ASPM localizes at the “minus ends” of microtubules, recruits katanin, and promotes katanin‐mediated severing of growing microtubules [13]. Given our finding of high ASPM expression levels in A549‐R cells, we reasoned that ASPM may participate in RT resistance by regulating microtubule dynamics. This notion was supported by our findings on immunofluorescence co‐localization analysis with α‐tubulin, γ‐tubulin, and acetylated α‐tubulin expressions (Figure 2A–C; Figure S1B), which revealed that ASPM binds along microtubules during interphase and co‐localizes with the spindle complex during mitosis. Acetylated α‐tubulin levels were also significantly lower in A549‐R cells than in A549 cells, indicating that stable microtubules had been destroyed in A549‐R cells. To gain further insight into the role of ASPM in A549‐R cells, we next examined its effects on microtubule formation during interphase. Immunofluorescence analysis revealed that ice‐induced microtubule depolymerization was exacerbated in A549‐R cells (Figure 2D). After microtubule depolymerization was complete, we incubated cells at 37°C to induce regrowth of microtubules, and we found that regrowth was consistently attenuated in A549‐R cells (Figure 2E). HeLa cells are widely used as a model system for studying various aspects of cell biology, including microtubule dynamics. Furthermore, HeLa cells have a well‐characterized karyotype, which can facilitate the interpretation of results obtained from studies of microtubule dynamics. We also found that irradiating HeLa cells stably expressing GFP‐α‐tubulin (HeLa‐TUBA) with 2 Gy led to substantial changes in microtubule length (Figure 2F–H). To substantiate the role of ASPM in microtubule stability, we knocked down ASPM expression in A549‐R cells and found that this knockdown led to increased microtubule acetylation. That is, microtubule stability was enhanced after ASPM was knocked down in A549‐R cells (Figure 2I,J). Collectively, these findings indicate that ASPM has a crucial role in regulating microtubule stability.

### 2.3 Spindle Misorientation in High‐ASPM‐Expressing RT‐Resistant Cells

**FIGURE 3:**
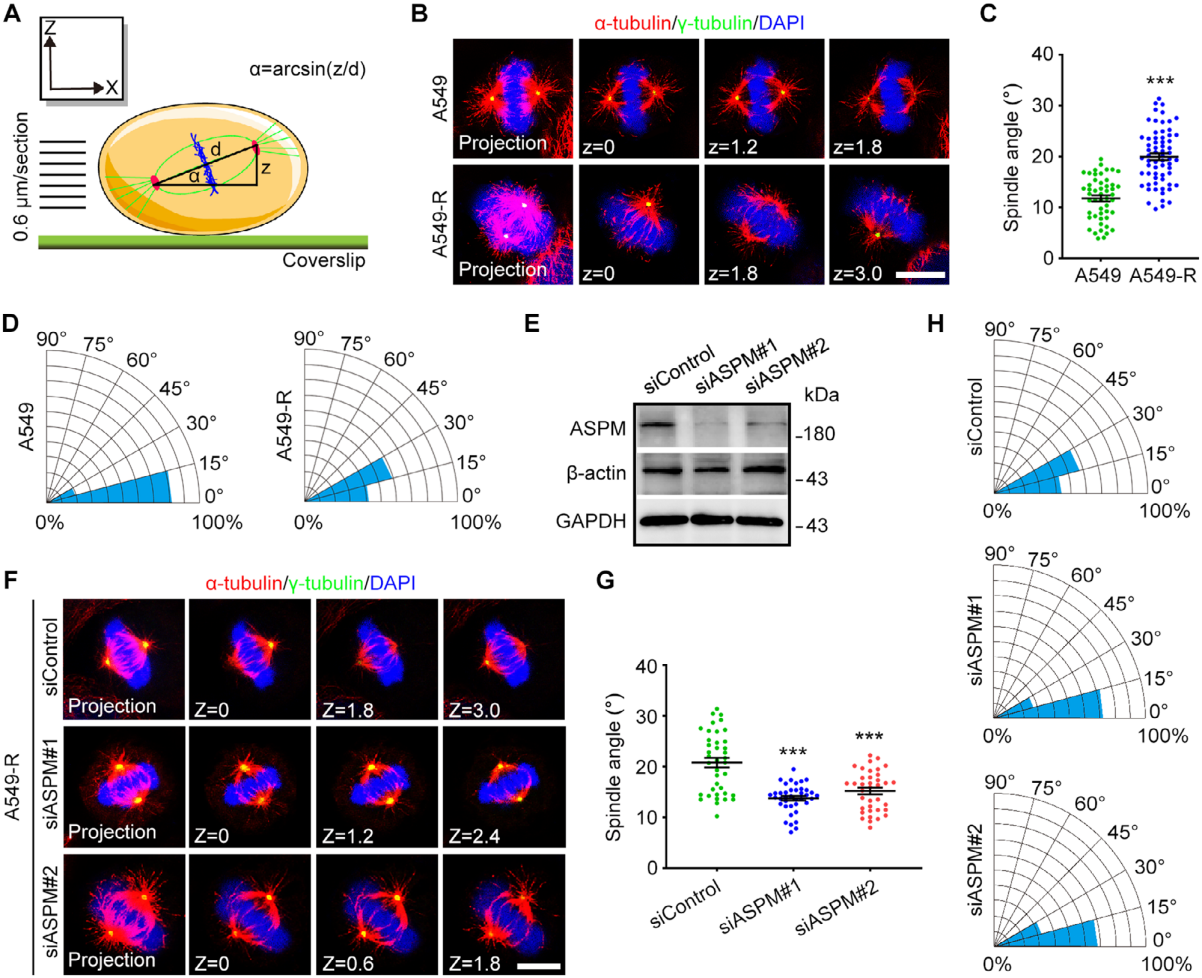
Loss of ASPM prevents spindle misorientation in A549‐R cells. (A) Schematic depicting the process for measuring spindle angles (α). Representative immunofluorescence images (B), spindle angles (C), and spindle angle distribution (D) of metaphase A549 cells and A549‐R cells stained with anti‐α‐tubulin (red) and anti‐γ‐tubulin (green) antibodies and DAPI (blue). Scale bar: 6 µm. *n* = 62 in A549 group. *n* = 59 in A549‐R group. (E) Immunoblots showing the effectiveness of *ASPM* siRNAs in A549‐R cells. Representative immunofluorescence images (F), spindle angles (G), and spindle angle distribution (H) of metaphase A549‐R cells transfected with control or *ASPM* siRNAs and stained with anti‐α‐tubulin (red) and anti‐γ‐tubulin (green) antibodies and DAPI (blue). Scale bar: 6 µm. ***p < 0.001. *n* = 35 in siControl group. *n* = 38 in siASPM#1 group. *n* = 42 in siASPM#1 group.

Stable microtubules are necessary for correct orientation of the mitotic spindles, which is important for ensuring the accuracy of subsequent chromosome segregation [15]. To investigate the adverse effects triggered by abnormal microtubule stability, we measured spindle orientation in A549‐R cells (Figure 3A). Fluorescence microscopy revealed that the spindles were misoriented in A549‐R cells (Figure 3B–D). To determine whether this misorientation could be reversed by downregulating ASPM, we introduced two different siRNAs to ASPM into A549‐R cells (Figure 3E) and found drastic changes in the spindle angles after siASPM treatment, with orientation returning to nearly that of non‐resistant A549 cells (Figure 3F–H). These functional assays demonstrated that high ASPM expression promoted spindle misorientation in lung cancer cells, which would be expected to negatively affect the equal segregation of chromosomes.

### 2.4 Chromosomal Mis‐Segregation Can Be Repaired by Suppressing ASPM Expression in RT‐Resistant Cells

**FIGURE 4:**
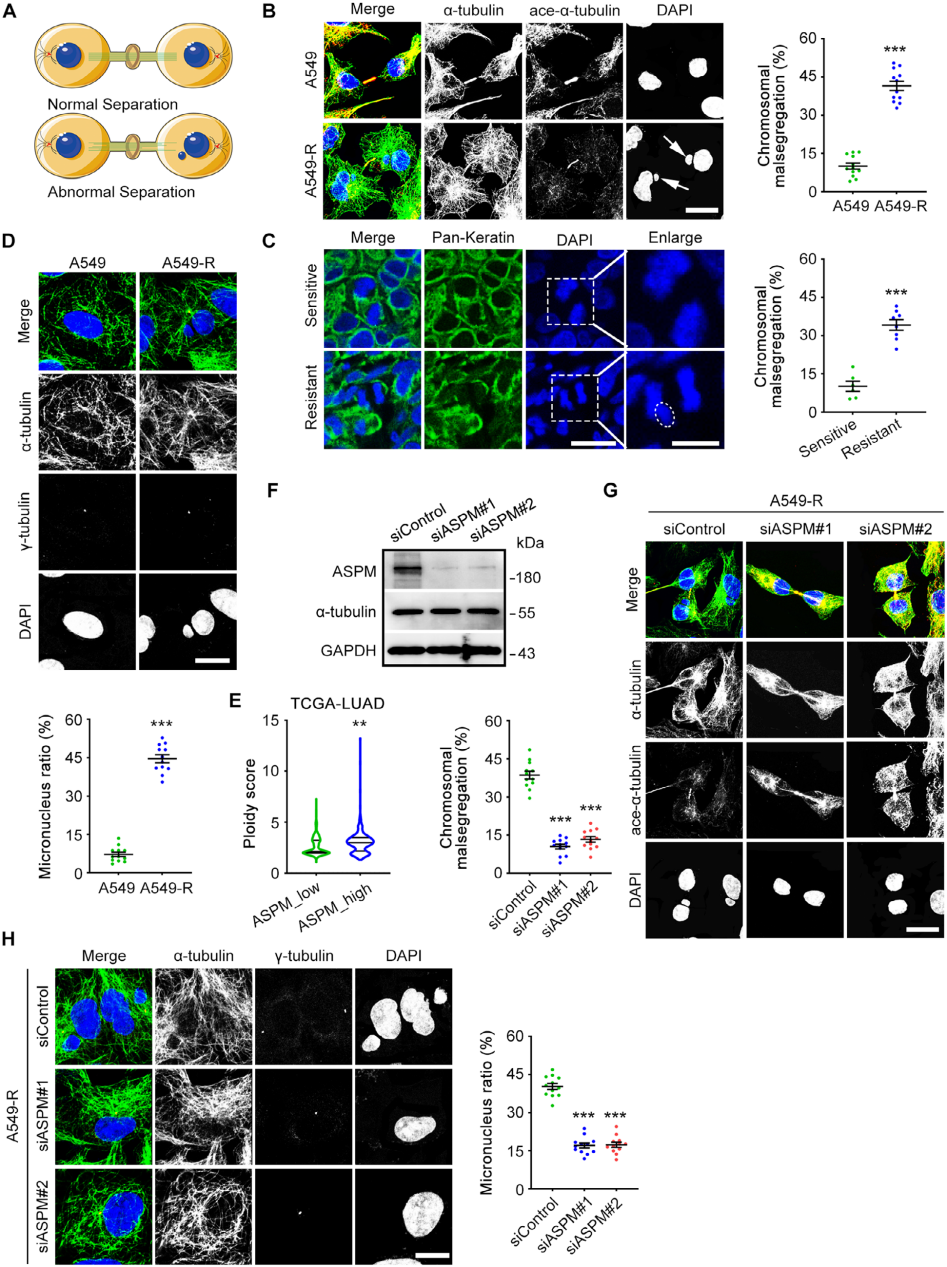
ASPM promotes incorrect chromosome segregation and formation of micronuclei. (A) Schematic diagram showing the effects of microtubule disruption during cytokinesis on chromosome mis‐segregation. (B) Immunofluorescence images of A549 cells and A549‐R cells during cytokinesis with anti‐α‐tubulin (green) and anti‐acetylated α‐tubulin (red) antibodies and DAPI (blue). Scale bar: 10 µm. *n* = 10 in A549 group. *n* = 12 in A549‐R group. (C) Fluorescence images of lung biopsy samples from patients with RT‐sensitive or RT‐resistant disease showing chromosomal segregation after metaphase of cancer cells with anti‐pan CK (green) and DAPI (blue), with quantification of chromosomal mis‐segregation. Scale bar: 20 µm (left) and 10 µm (right). *n* = 6 in Sensitive group and *n* = 8 in resistant group. (D) Immunofluorescence images of A549 cells and A549‐R cells with anti‐α‐tubulin (green) and anti‐γ‐tubulin (red) antibodies and DAPI (blue) showing generation of micronuclei in the cells, with quantification of micronuclei. Scale bar: 10 µm. *n* = 12 in each group. (E) Ploidy score showing the correlation between ASPM expression and generation of ploidy, analyzed with The Cancer Genome Atlas database. (F) Immunoblots showing the effectiveness of *ASPM* siRNAs in A549‐R cells. (G) Immunofluorescence images of A549‐R cells with control or *ASPM* siRNAs treated and stained with anti‐α‐tubulin (green) and anti‐acetylated α‐tubulin (red) antibodies and DAPI (blue) during cytokinesis, with quantification of chromosomal mis‐segregation. Scale bar: 10 µm. *n* = 12 in each group. (H) Immunofluorescence images of A549‐R cells transfected with control or *ASPM* siRNAs, followed by staining with anti‐α‐tubulin (green) and anti‐γ‐tubulin (red) antibodies and DAPI (blue) showing generation of micronuclei in the cell, with quantification of micronuclei. Scale bar: 10 µm. **p < 0.01. ***p < 0.001. The experiment was repeated twelve times, at least 36 cells in each group.

Next, to understand the downstream effects of misoriented spindles and how they might contribute to RT resistance in cancer cells, we studied chromosome segregation conditions during cytokinesis. As noted previously, failures in cytokinesis lead to increased genomic instability, which increases the likelihood of chromosome combinations that may provide a selective growth advantage (Figure 4A). Consistent with this notion, we saw substantial increases in chromosomal mis‐segregation in A549‐R cells relative to A549 cells (Figure 4B). Importantly, chromosomal mis‐segregation has also been observed in samples from patients with RT‐resistant NSCLC (Figure 4C), which were verified as taking place in individual cells through co‐staining with γ‐tubulin (Figure 4D). In the meantime, we calculated ploidy scores for lung adenocarcinoma samples from The Cancer Genome Atlas dataset and found that high ASPM expression was more likely to be linked with high ploidy scores (Figure 4E). To further examine this observation, we knocked down ASPM in A549‐R cells and found drastic reductions in chromosomal mis‐segregation and fewer micronuclei (Figure 4F–H). Given the common presence of high ASPM expression in A549‐R cells, we next proposed that ASPM can disrupt microtubule dynamics and affect genomic instability by offering RT‐resistant cells the possibility to establish an “escape route” to survive.

### 2.5 Knocking Down ASPM in RT‐Resistant Cells Promotes RT‐Induced Apoptosis

**FIGURE 5:**
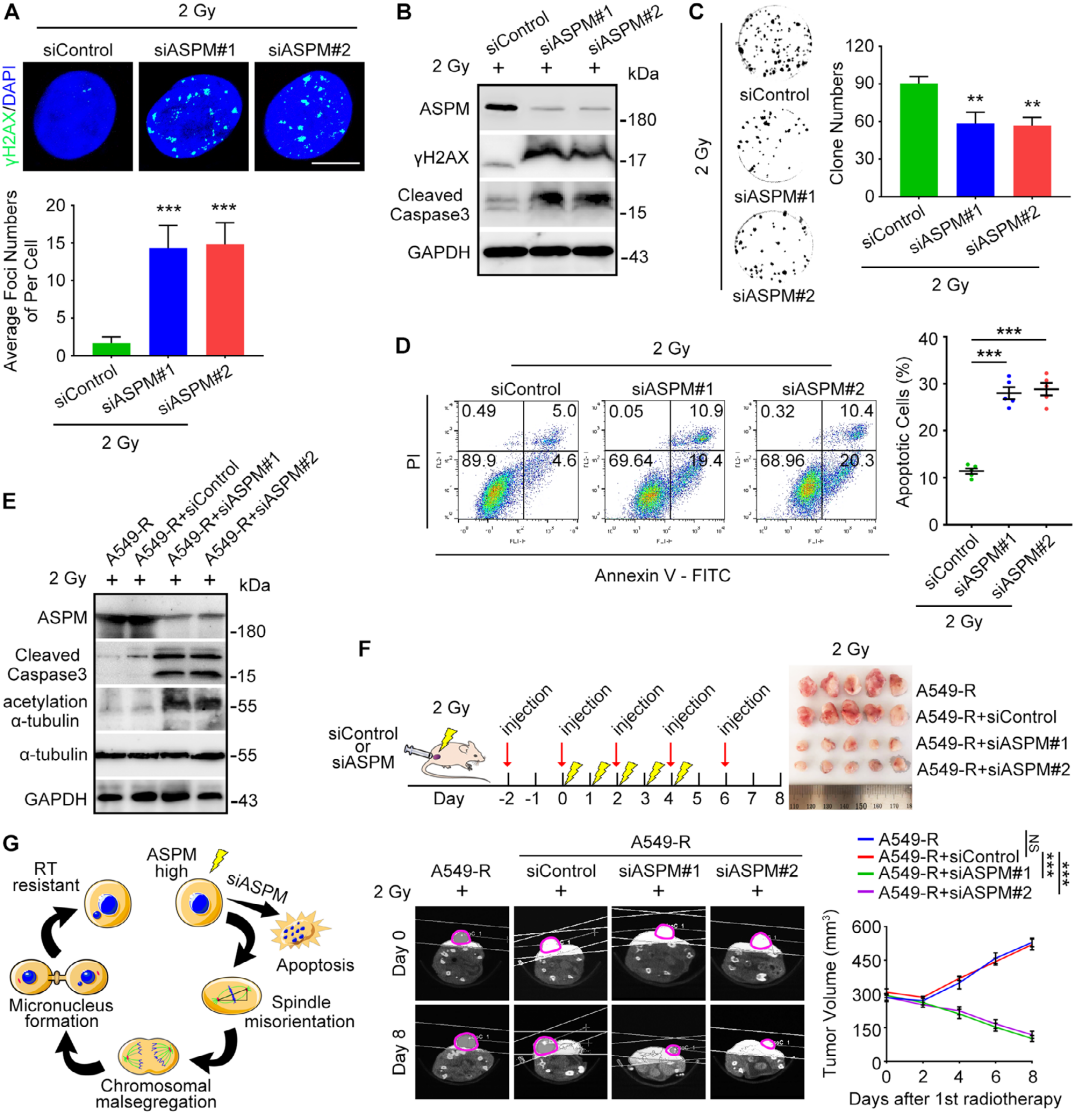
ASPM depletion re‐sensitizes RT‐resistant cells to RT‐induced apoptosis. (A) Immunofluorescence images of γH2AX foci in A549‐R cells treated with control or *ASPM* siRNAs followed by irradiation to 2 Gy, with quantification of foci per cell. Scale bar: 8 µm. The experiment was repeated three times, 30 cells in each group. (B) Immunoblots showing levels of ASPM, γH2AX, cleaved caspase‐3, and β‐actin in A549‐R cells transfected with control or *ASPM* siRNAs followed by treatment with 2 Gy. (C) Representative analyses of numbers of A549‐R clones treated with control or *ASPM* siRNAs and then treatment with 2 Gy. (D) Flow cytometry analysis of apoptotic A549‐R cells treated with control or *ASPM* siRNAs followed by treatment with 2 Gy. The experiment was repeated five times in each group, *n* = 5. (E) Immunoblots showing levels of ASPM, cleaved caspase‐3, acetylated α‐tubulin, α‐tubulin, and β‐actin in subcutaneous tumors from nude mice implanted with cells incorporating control‐ or *ASPM*siRNAs after treatment with 2 Gy. (F) Subcutaneous tumor volumes formed by A549‐R cells treated with *ASPM* siRNAs were reduced after treatment with 2 Gy. Five mice in each group, *n* = 5. NS, not significant; **p < 0.01. ***p < 0.001. The experiment was repeated three times, 6 mice in each group. (G) Schematic diagram showing the consequences of irradiating RT‐resistant cells with high or low expression of ASPM.

Our findings thus far support the notion that ASPM affects genomic instability in A549‐R cells. Meanwhile, knock down ASPM expression decreased cells proliferation both in vitro and in xenografts (Figure S3). Although the importance of genomic stability in cancer development and progression is well established [16], the question of whether downregulating ASPM expression can increase apoptosis in RT‐resistant cells such as A549‐R remains unanswered. To address this question, we analyzed apoptosis in A549‐R cells treated with siRNA to ASPM. Notably, downregulating ASPM affect apoptosis in A549 cells (Figure S2A) but not in A549‐R cells (Figure S4). However, when siASPM cells were treated with 2 Gy irradiation, γH2AX expression was significantly increased relative to the non‐irradiated group (Figure 5A,B), leading us to analyze apoptosis again under these conditions. Intriguingly, cleaved caspase‐3 expression was increased and numbers of clones were decreased compared with unirradiated RT‐resistant cells (Figure 5B,C), indicating a sharp increase in numbers of apoptotic cells. These results were confirmed by flow cytometry (Figure 5D; Figures S1C and S2B). We further collected subcutaneous tumors from mice implanted with ASPM‐siRNA tumor cells and treated with 2 Gy and analyzed the expression of acetylated α‐tubulin and cleaved caspase‐3 by Western blotting. Consistent with the findings from the cultured siASPM‐treated cells, expression of both acetylated α‐tubulin and cleaved caspase‐3 increased (Figure 5E). Moreover, tumor volumes and apoptotic marker were significantly smaller in the mice with siASPM A549‐R implants treated with 2 Gy (Figure 5F; Figure S5). Collectively, these findings show that ASPM has similar effects in vivo and in vitro and suggest that ASPM in RT‐resistant cells has a conserved role in the pathogenesis and progression of lung adenocarcinoma by affecting the stability of microtubules (Figure 5G).

### 2.6 Targeting the 963–1263 Amino Acid Region of ASPM as Anticancer Therapy

**FIGURE 6:**
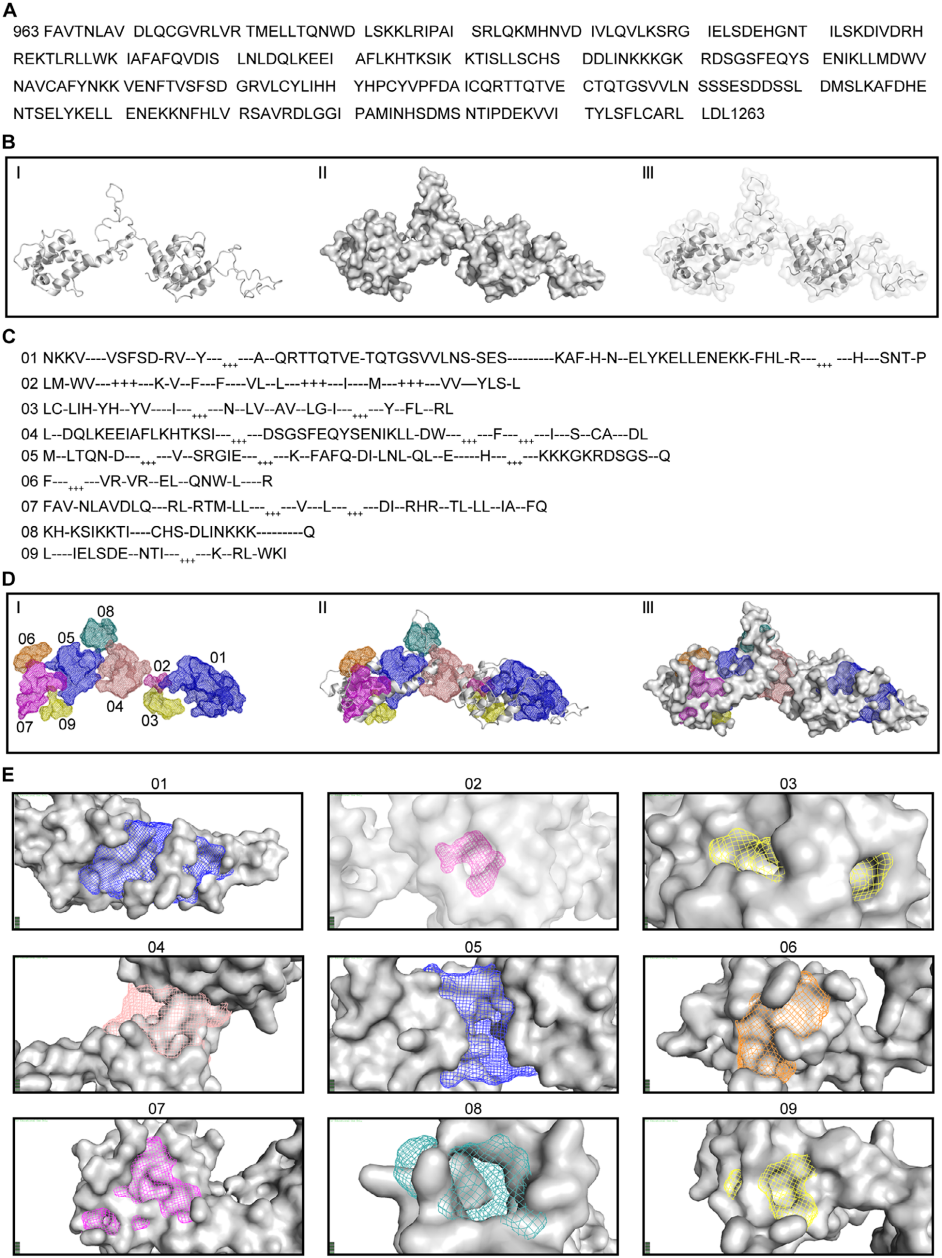
Targeting amino acid region 963–1263 of ASPM as a new strategy to overcome RT resistance. (A) Illustration of amino acids 963–1263 in human ASPM. (B) Homologous structure model of human ASPM acid amino region 963–1263 based on bioinformatics analysis. Images showing carbon structure (I), surface structure (II), and the overlay (III). (C) Nine drug pockets corresponding to the acid amino region sequence of ASPM. (D) Images showing the regions of the nine drug pockets in ASPM. (I) Pocket shapes; (II) pockets on ASPM carbon structure; and (III) pockets on the ASPM surface structure. (E) Magnification of each of the nine drug pockets on ASPM surface structure.

The anticancer drug paclitaxel and its analogues act by directly targeting and stabilizing microtubules; however, this strategy can still result in drug resistance [17]. To circumvent this problem, we explored potential drug targets within the ASPM protein as follows. First, we searched the SWISS‐MODEL database for homologous sequences of ASPM and found its 963–1263 amino acid sequences to be highly matched to integrin alpha‐L structure (MMDB ID: 29803) (Figure 6A). We next created model structures based on this region (Figure 6B) and discovered 9 candidate drug–target sequences there (Figure 6C). We then used this information to predict the location of “drug pockets” and to estimate their shapes and sizes. We found 9 such drug pockets for ASPM by using this strategy (Figure 6D,E). At this time, we speculate that these regions participate in the inhibition of ASPM in RT‐resistant cancer cells. We are currently constructing different truncated mutations of ASPM to identify the functional region(s) responsible for RT resistance in future studies. In the meantime, we are actively developing a new small‐molecule inhibitor of ASPM for treating RT‐resistant disease, an effort that is expected to have important implications for improving the diagnosis and treatment of patients with RT‐resistant NSCLC.

## 3 Discussion

The major cause of failure of clinical cancer therapy was RT resistance. Hence, clarifying the mechanisms of RT resistance could help patients who had RT‐resistant disease avoid expensive medical expenses of ineffective treatment. Though some new methods were used to treat cancer therapy, much work remains to be done for considerably enhanced therapeutic efficacy [18]. The growth trend of ASPM for tumor treatment has led us to examine its potential involvement in the therapeutic response to RT‐resistant cancer [19]. In the present study, we used in vitro cell culture models, in vivo mouse models, and analysis of human genome databases to clarify the importance of ASPM in radiation resistance in NSCLC. We found that ASPM has a crucial role in RT resistance by affecting microtubule stability and chromosomal mis‐segregation during mitosis. Indeed, high expression of ASPM correlated directly with chromosome mis‐segregation and increased numbers of micronuclei, suggesting that high expression of ASPM in NSCLC is more likely to be intrinsic and integral to RT resistance rather than incidental. This notion is also supported by recent comprehensive functional analyses of ASPM in different types of tumors in patients [20]. We propose that mis‐segregation of chromosomes could have much broader effects extending beyond radiation resistance in cancer, which underscores the broad clinical value of understanding the exact role of the genomic instability in cancer progression and therapy.

Regulation of microtubule dynamics is a crucial aspect of a wide variety of physiological and pathological processes [21]. Abnormal microtubule dynamics in cells represent an obstacle to normal physiological activity. Many microtubule‐associated proteins can affect microtubule dynamics. Any promotion of anomalies in microtubule dynamics via changes in genes or microtubule‐associated proteins can have uncertain results that may have catastrophic consequences to organisms [22]. Although some drugs currently in clinical use such as paclitaxel and docetaxel can directly target microtubules, such drugs can also lead to or worsen drug resistance. Our findings provide a new target strategy for RT‐resistant NSCLC cells—that is, to use ASPM inhibition to decrease micronucleus formation by stabilizing microtubule dynamics during mitosis. Moreover, this mechanism is not restricted to NSCLC. Because of high ASPM levels have been linked with tumor development and progression in colorectal cancer as well [23]. Therefore, ASPM could be a target molecule for preventing RT resistance in a broad range of cancer types, prevention being a much better alternative than having to treat resistant cancer after it develops.

In the future, targeting ASPM may have biological consequences distinct from targeting microtubules upon the appearance of RT‐resistant tumor cells. Importantly, it may circumvent the problem of developing resistance to commonly used chemotherapeutic agents. Inoperable patients can check ASPM expression to choose a better clinical treatment to prevent radiotherapy resistance occurrence. ASPM has also been implicated in promoting homologous recombination‐mediated DNA repair [24], adding another layer of evidence in resistance to radiation. This may explain, at least in part, why cancer cells can evade spindle checkpoint monitoring. Abnormal microtubule dynamics in RT‐resistant cells can have a variety of consequences, leading to complex outcomes. Given our positive results regarding the involvement of ASPM in RT‐resistant cancer cells, we forecast its effectiveness as suggested by homology modeling. Future in‐depth analyses of why ASPM expression differs, how to specifically inhibit it, and the development of ASPM‐specific drugs will provide insights on how best to implement strategies for accurate diagnosis and selection patients for treatment of RT‐resistant cancer.

## 4 Materials and Methods

### 4.1 Materials

Antibodies targeting α‐tubulin (ab18251 and ab7291), γ‐tubulin (ab11316 and ab11317), and Alexa Fluor‐conjugated secondary antibodies were purchased from Abcam (Cambridge, UK). Antibodies targeting cleaved caspase‐3 (9664S), γH2AX (9718S), and pan‐keratin (4545) were purchased from CST (Danvers, MA). Antibodies targeting ASPM (26223‐1‐AP), β‐actin (20536‐1‐AP), and acetylated α‐tubulin (66200‐1‐Ig) were purchased from ProteinTech (Rosemont, IL). Horseradish peroxidase‐conjugated secondary antibodies were from Santa Cruz Biotechnology (Santa Cruz, CA). 4ʹ,6‐diamidino‐2‐phenylindole (DAPI) was obtained from Sigma‐Aldrich. Small interfering RNAs targeting *ASPM* (#1: 5ʹ‐GCAGCATGCCGTTTGTTTA‐3ʹ; #2: 5ʹ‐CAGAGATGGTTTCGAGCAA‐3ʹ) for in vitro and in vivo experiments were synthesized by RiboBio (Guangzhou, China).

### 4.2 Cell Culture and Treatment

A549 cells were obtained from the American Type Culture Collection. HeLa cells stably expressing GFP‐α‐tubulin (HeLa‐TUBA) were obtained as we described previously [15a]. Cells were cultured in DMEM (Thermo Fisher Scientific, Waltham, MA) supplemented with 10% fetal bovine serum (FBS). siRNAs were transfected with Lipofectamine RNAimax (Thermo Fisher Scientific). Cell cultures were irradiated with an RS2000 Pro‐225 Biological X‐ray irradiator (RadSource, Buford, GA). After 8 h of the irradiation, non‐adherent cells were removed and washed twice with PBS. Adherent cells were further incubated in Dulbecco's modification of Eagle's medium (DMEM) containing 10% FBS, 100 µg/mL penicillin, and 100 U/mL streptomycin (Invitrogen, NY) for 16 h until 80%–90% confluence in 5% CO_2_ at 37°C. After 24 h of the irradiation, cells were treated by trypsin. The suspension was neutralized with complete medium and centrifuged at 2000 rpm for 5 min. The cell pellet was suspended in DMEM containing 10% FBS, 100 µg/mL penicillin, and 100 U/mL streptomycin (Invitrogen, NY). The cells were renewed grown in the culture dishes.

### 4.3 Animal Experiments

Nude mice were obtained from Beijing Huafukang Biotechnology Development Co., Ltd. (Beijing, China). A549 and A549‐R cells were injected subcutaneously into the flanks of the mice and allowed to form tumors. When subcutaneous tumor volumes reached about 300 mm^3^, the mouse tumors were irradiated with 2 Gy by using a Small Animal Radiation Research Platform (Xstrahl, UK). *siControl* and *siASPM* injected siRNAs in vivo were designed and synthesized from Ribobio (Guangzhou, China), the study protocol was described as previously [25]. All applicable institutional and/or national guidelines for the care and use of animals were followed. The use of mice was approved by the Animal Care and Use Committee of Shandong Cancer Hospital and Institute (SDTHE C2024003162). BALB/c mice were used in experiment, at least 5 mice in each group. 1 × 10^6^ cells were inoculated subcutaneously into nude mice to develop implant tumor in each mice.

### 4.4 Fluorescence Microscopy

Permeabilized (with 0.5% Triton X‐100) grown cells for experimentation were fixed on coverslips with 4% paraformaldehyde, and 2% bovine serum albumin were used to block the cells. Then, cells were sequentially probed with the primary antibodies, fluorophore‐conjugated secondary antibodies, and DAPI. The coverslips were mounted with mounting medium and images obtained with an LSM 800 confocal microscope (Zeiss, Oberkochen, Germany). Spindle angles were measured as described previously [26]. Number of clones and γH2AX foci were counted with ImageJ software. For time‐lapse microscopy, mitotic progression was recorded with a Cell Observer 7 live‐cell optical fault microscopic monitoring system with a live‐cell chamber as described method [27].

### 4.5 Immunoblotting Analysis

Cells were first lysed, using cell lysis buffer (Beyotime Biotech., China), and then mixed with sodium dodecyl sulfate (SDS) sample buffer. Targeted proteins were visualized with an enhanced chemiluminescence substrate kit. First, the proteins were resolved by gel (10% SDS–polyacrylamide) electrophoresis before being transferred to the PVDF (polyvinylidene fluoride) membranes (Millipore), which were blocked in Tris‐buffered saline (containing 0.1% Tween 20 and 5% fat‐free milk). Then, primary antibodies and secondary antibodies (horseradish peroxidase conjugated) were sequentially incubated with the membranes.

### 4.6 Apoptosis Assays

To verify apoptosis, flow cytometry (BD Biosciences) was used to examine stained cells (using FITC Annexin‐V apoptosis detection kit). And LSM 800 confocal microscope (Zeiss) was also used to examine stained cells (using terminal deoxynucleotidyl transferase‐mediated dUTP nick end labeling [TUNEL] apoptosis detection kit).

### 4.7 Statistical Analysis

Prism 8 (GraphPad) was used for data plotting and statistical analysis. All experiments were repeated independently at least three times, and data are expressed as means ± standard error of the mean (SEM). Student's *t*‐tests were used for pairwise comparisons, and analysis of variance (ANOVA) for comparison of multiple groups, with the built‐in analysis tools of Prism 8. Normal distribution, variations within each group, and sample independence were assumed to be met. All measurements were taken from distinct samples, and no data were excluded. *p* values are denoted by asterisks as: *p < 0.05; **p < 0.01; ***p < 0.001, with *p* < 0.05 considered to indicate statistical significance.

## Author Contributions

Tao Zhong, Liewei Wen, and Jinming Yu conceived and designed the study. Fei Wu and Minglei Wang performed the bioinformatic analysis. Tao Zhong, Juan Wang, Songbo Xie, Changyan Xiao, Meng Wu, Xiaoxiao Gongye, Lisheng Liu, and Xiaozheng Chen performed the experiments. Tao Zhong, Ning Liu, and Dawei Chen wrote the paper. All authors reviewed the manuscript.

## Ethics Statement

We are highly obliged to patients for their cooperation and participation in this study. The study protocol obtained approval from the Ethics Committee of Shandong Cancer Hospital and Institute (Animal SDTHE C2024003162; Human SDTHEC2021006008). Written informed consent for presentation and publication of this study was obtained from all the patients.

## Conflicts of Interest

The authors declare no conflicts of interest.

## Supporting information



Supporting Information

## Data Availability

The data that support the findings of this study are available from the corresponding author upon reasonable request.
