# Supplementary material for: ASPM Induces Radiotherapy Resistance by Disrupting Microtubule Stability Leading to Chromosome Malsegregation in Non‐Small Cell Lung Cancer
Source: Exploration (Beijing). 2025 May 7;5(4):e20230024. doi: 10.1002/EXP.20230024 (PMC12380063; doi:10.1002/EXP.20230024)
Supplement: Supplementary file 1 — Supporting Information [file EXP2-5-e20230024-s001.docx]

**Supplementary Material**


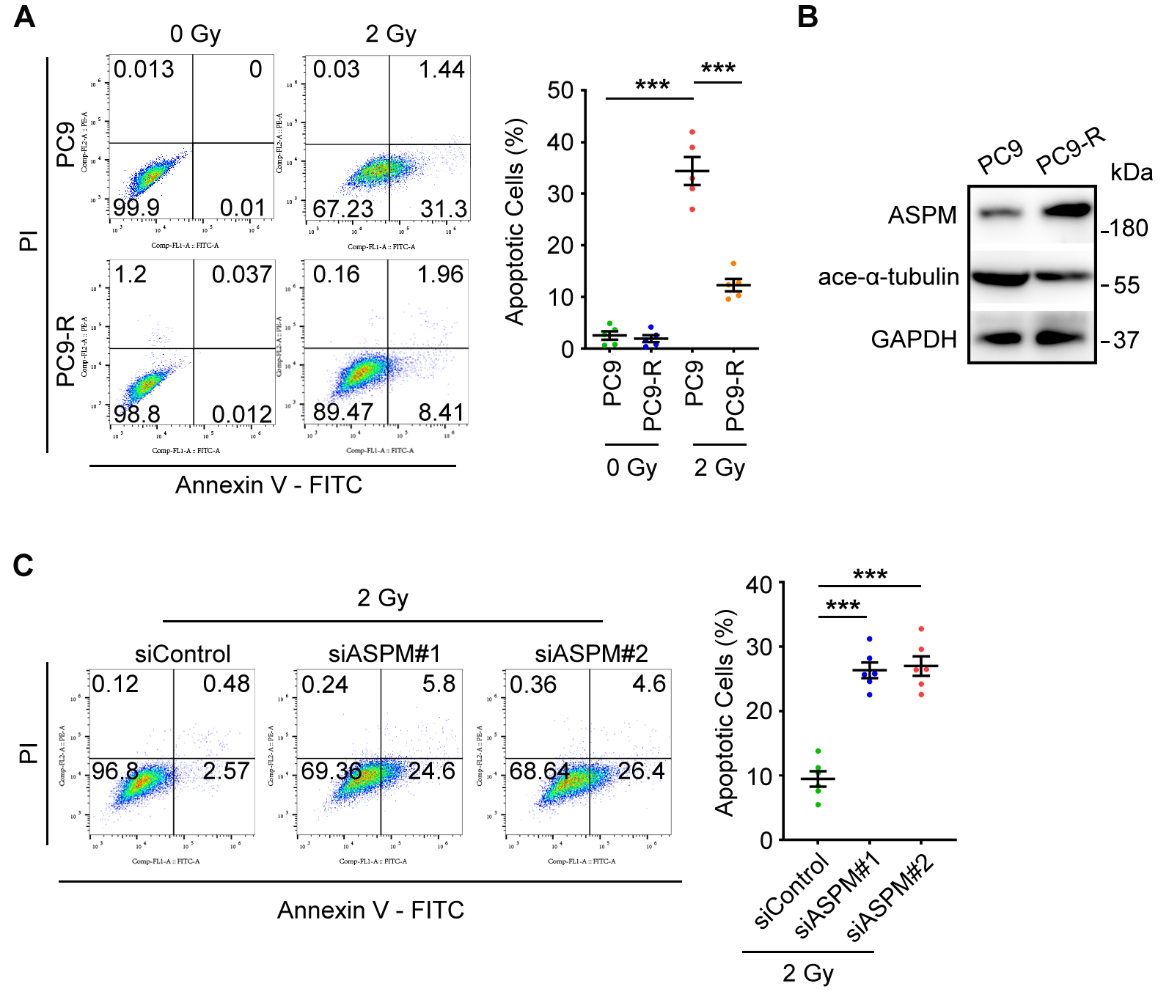


**Fig S1. ASPM deficiency promotes the apoptosis rate of RT-Resistant PC9-R cells.** (A) Flow cytometry analysis of apoptotic cell percentages in PC9 cells and PC9-R cells after irradiation to 2 Gy. The experiment was repeated five times in each group, n=5. (B) Immunoblots showing levels of acetylated α-tubulin in PC9 cells and PC9-R cells. (C) Flow cytometry analysis of apoptotic PC9-R cells treated with control or *ASPM* siRNAs followed by treatment with 2 Gy. The experiment was repeated six times in each group, n=6. ****P* < 0.001.


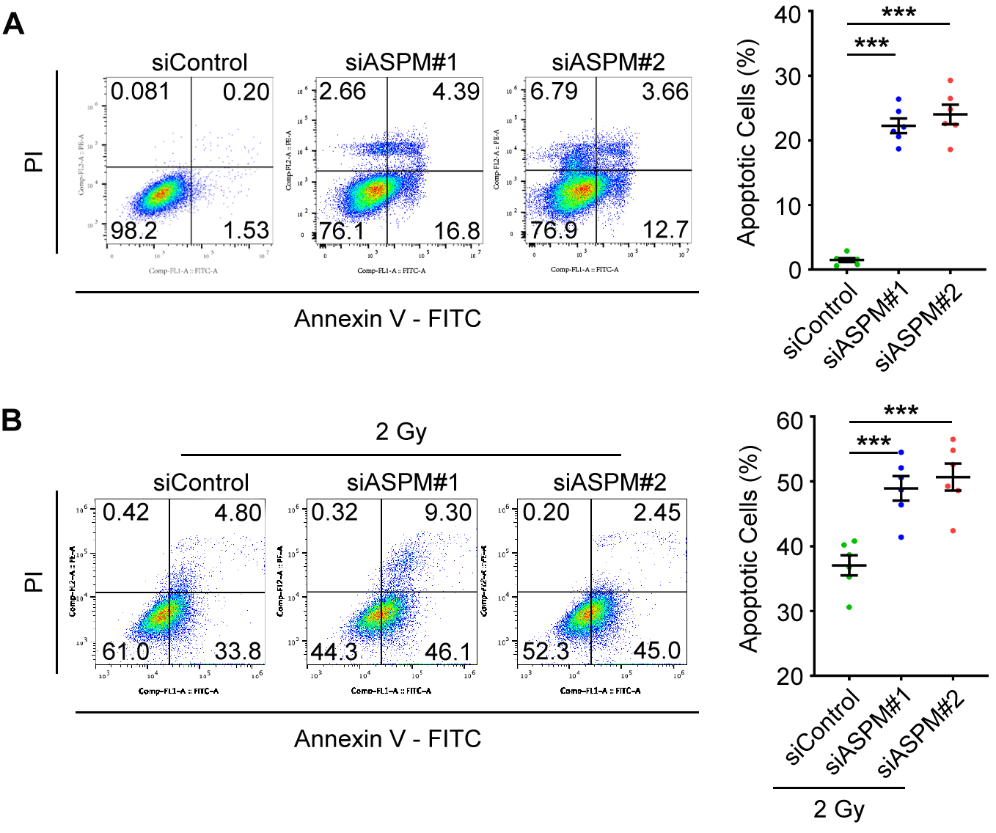


**Fig S2. ASPM depletion increase the radiosensitivity of the parental A549 cells.** (A) A549 cells transfected with siControl or *ASPM* siRNAs were stained with Annexin V-FITC and propidium iodide, and apoptotic cells were detected by flow cytometry. The experiment was repeated six times in each group, n=6. (B) Flow cytometry analysis of apoptotic A549 cells treated with control or *ASPM* siRNAs followed by treatment with 2 Gy. The experiment was repeated six times in each group, n=6. ****P* < 0.001.


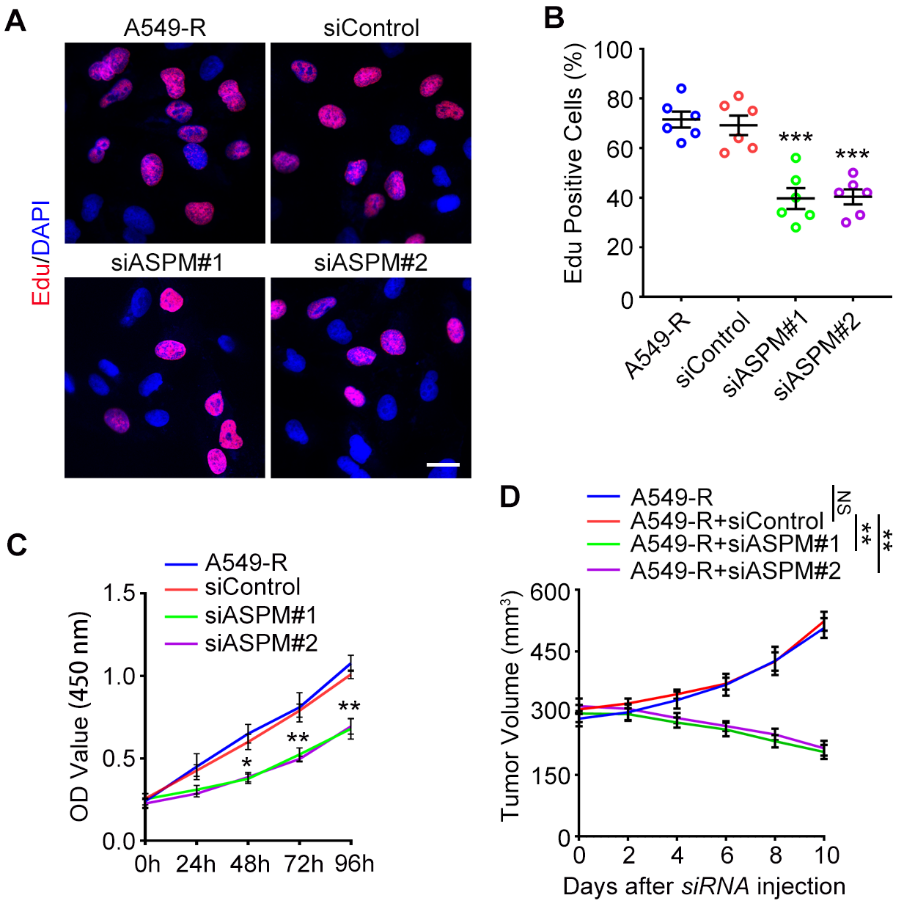


**Fig S3. Loss of ASPM prevents the proliferation in A549-R cells and in xenografts.** (A and B) The proliferation rate of A549-R cells transfected with control or *ASPM* siRNAs were determined by EdU staining. Scale bar, 10 μm. The experiment was repeated six times in each group, 10 cells in each group. (C) A549-R cells were treated with control or *ASPM* siRNAs for the indicated time, and the extent of cell proliferation and viability was determined by MTT assays. The experiment was repeated three times in each group, n=3. (D) Subcutaneous tumor volumes formed by A549-R cells were reduced after treatment with *ASPM* siRNAs. The experiment was repeated three times, 5 mice in each group. NS, Not Significant; **, *P <* 0.01; ***, *P <* 0.001.


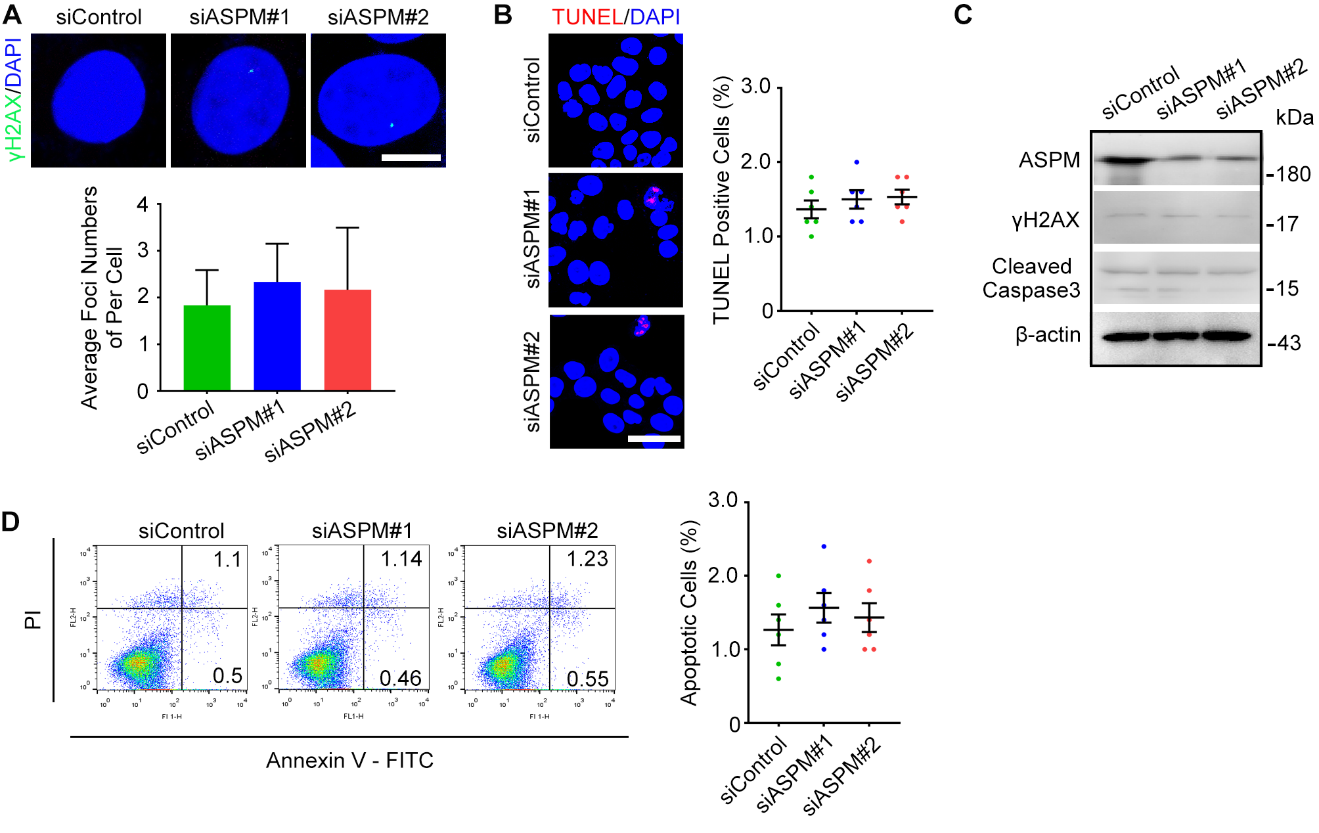


**Fig S4. ASPM deficiency does not affect apoptosis in A549-R cells.** (A) Immunofluorescence staining and quantification of γH2AX foci in A549-R cells treated with control or *ASPM* siRNAs. Scale bar, 8 μm. n=12 in each group. (B) A549-R cells transfected with siControl or *ASPM* siRNAs were stained with TUNEL to identify apoptotic cells. Scale bar, 20 μm. The experiment was repeated six times in each group, n=6. (C) A549-R cells were transfected with siControl or *ASPM* siRNAs, and protein levels were determined by immunoblotting with the indicated antibodies. (D) A549-R cells transfected with siControl or *ASPM* siRNAs were stained with Annexin V-FITC and propidium iodide, and apoptotic cells were detected by flow cytometry. The experiment was repeated six times in each group, n=6.


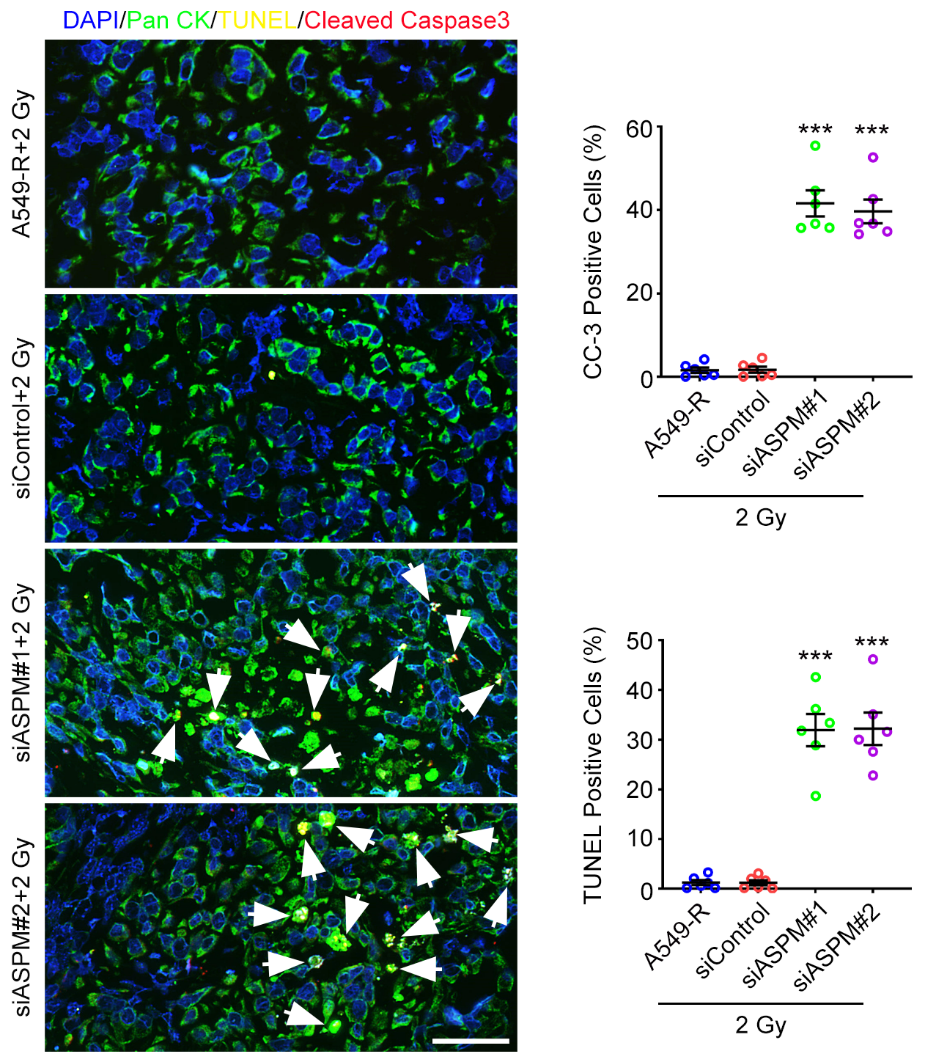


**Fig S5. ASPM deficiency promotes the apoptosis of A549-R cells xenografts tumor with 2 Gy irradiation.** The positive cells rate of A549-R cells injected with control or *ASPM* siRNAs after irradiation *in vivo* were determined by immunofluorescent staining with anti-Cleaved Caspase3 (red) and anti-Pan CK (green) antibodies, TUNEL (lemon yellow) and DAPI (blue). Cleaved Caspase3 and TUNEL co-stain cells were indicated by white arrows. 6 mice in each group, n=6. Scale bar, 50 μm. ***, *P* < 0.001.
